# Hard tissue stability after guided bone regeneration: a comparison between digital titanium mesh and resorbable membrane

**DOI:** 10.1038/s41368-021-00143-3

**Published:** 2021-11-16

**Authors:** Songhang Li, Junyi Zhao, Yu Xie, Taoran Tian, Tianxu Zhang, Xiaoxiao Cai

**Affiliations:** State Key Laboratory of Oral Diseases & National Clinical Research Center for Oral Diseases & Department of Implant dentistry, West China Hospital of Stomatology, Sichuan University, Chengdu, China

**Keywords:** Dental diseases, Translational research

## Abstract

Guided bone regeneration (GBR) uses resorbable and non-resorbable membranes as biological barriers. This study compared the differences in hard tissue stability between GBR using evidence-based digital titanium mesh and resorbable collagen membranes during implant placement. A total of 40 patients (65 implant sites) were enrolled and divided into two groups: resorbable membrane and digital titanium mesh groups. The alveolar bone was analyzed at two- and three-dimensional levels using cone-beam computed tomography and by reconstructing and superimposing the hard tissues at four time points: preoperatively, postoperatively, before second-stage surgery, and 1 year after loading. The use of digital titanium mesh showed less alveolar bone resorption in vertical and horizontal directions two-dimensionally before the second-stage surgery and 1 year after loading. Regarding volumetric stability, the percentage of resorption after 6 months of healing with resorbable membrane coverage reached 37.5%. However, it was only 23.4% with titanium mesh. Although postoperative bone volume was greater at all labial sites with resorbable membrane than with digital titanium mesh, after substantial bone resorption within 1 year of loading, the labial bone thickness at the upper part of implants was thinner with resorbable membrane than with digital titanium mesh. Furthermore, digital titanium meshes made according to ideal bone arch contour reduced soft tissue irritation, and the exposure rate was only 10%. Therefore, although both resorbable membrane and digital titanium mesh in GBR were able to successfully reconstruct the bone defect, digital titanium meshes were better at maintaining the hard tissue volume in the osteogenic space.

## Introduction

Tooth loss may result in alveolar atrophy due to bone resorption, which often leads to the loss of soft and hard tissues and affects facial profile in patients.^1^ Owing to this, it is not always possible to obtain sufficient bone volume around the implant during implant restoration, ultimately making it difficult to achieve a satisfactory outcome.^2,3^ Guided bone regeneration (GBR) is a common clinical solution for localized hard tissue defects, has been well documented as a successful method for reconstructing maxillary and mandibular bone defects.^4–8^ In GBR, a barrier membrane is used to artificially establish a biological barrier that prevents fibroblasts and epithelial cells from rapidly proliferating and entering the bone defect and gives priority to the growth of osteoblasts and blood vessels, thereby avoiding competitive inhibition between other cells and osteoblasts.^9^

Therefore, the purpose of GBR is to allow the preferential entry of osteoblasts into the osteogenic space through a barrier membrane and the formation of new bone in the osteogenic space using bone replacement materials as a scaffold, thus making a stable osteogenic space.^10^ If the osteogenic space is not sufficiently stable, it will collapse under external tissue pressure, resulting in a displacement of the bone grafts and failure to achieve the desired clinical outcome.^11,12^ Thus, the ideal material for barrier membrane in GBR requires a balance of good biocompatibility and excellent support.^13^ Resorbable collagen membranes are a popular choice for clinical treatment because of their low surgical complication rate and lack of need for secondary surgical removal. However, their soft consistency and poor mechanical properties often lead to compression of the osteogenic space and loss of tissue volume during new bone formation.^14^ For example, Schwarz et al. in a study using an animal model treated with GBR using a collagen membrane reported that although the bone defect was filled with bone graft material and covered with collagen membrane, the collagen membrane and bone graft material were displaced in the apical direction after 9 weeks.^15^ Compared to resorbable collagen membranes, titanium meshes have excellent mechanical properties, a higher spatial maintenance ability, and therefore, can provide a more stable osteogenic space.^16^

In our previous study, we found that a titanium mesh acted as a suitable barrier membrane and facilitated the development of good bone volume around an implant at the 41-month follow-up, confirming its excellent spatial maintenance ability.^17^ According to the concept of “prosthetically guided regeneration”, first proposed by Castentini et al., tissue regeneration is dependent upon the ideal positioning of the prosthesis. Inspired by the above-mentioned study and concept, we designed a digital titanium mesh to guide bone augmentation according to the position of the prosthesis (which determined the position of the implant).^18^ To achieve this, a digital smile design program was used to establish the ideal position of the prosthesis.^19,20^ After obtaining the patients’ intraoral scanning data and digital imaging and communications in medicine (DICOM) data, the implant was placed in the ideal three-dimensional position, and then the alveolar bone around the implant was reconstructed digitally using the software according to evidence-based guidelines, following which a reconstructed model was printed.^21^ The titanium mesh was preformed on the printed bone model, autoclaved, and then prepared for intraoperative use.^22^ Preoperative preparation of the mesh greatly reduced the operative time, patient’s pain, and the possibility of infection in the operative area. In addition, we reduced the mechanical stimulation by the titanium mesh to the soft tissues by covering it with a collagen membrane and by the purposeful design of the bone augmentation area.^23^ Moreover, in our previous study, a lower exposure rate was observed for digital titanium meshes compared with other studies.^17,22^ Therefore, the aforementioned methods could be utilized to overcome the disadvantages of the titanium mesh to obtain optimal osteogenic results, minimal complication rates, and good spatial maintenance.^24^

**Fig. 1:**
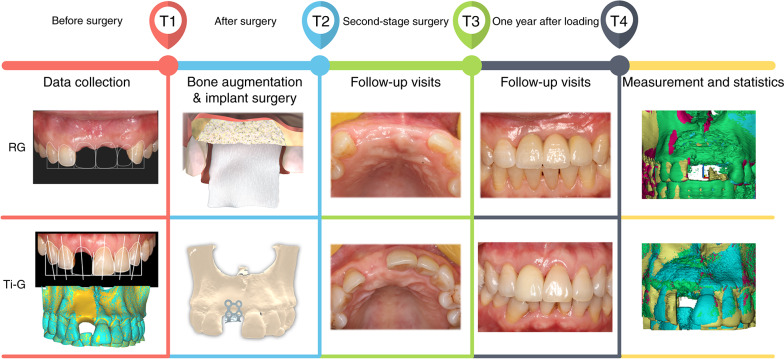
Treatment process and key time points. (T1, before surgery; T2, after surgery; T3, before second-stage surgery, T4, 1 year after loading)

The null hypothesis, which was no difference between the digital titanium meshed and the resorbable membranes in maintaining hard tissue stability after GBR, was applied in this study. Subsequently, by observing the changes in the peri-implant hard tissue at two-and three-dimensional levels at multiple time points (T1: preoperatively, T2: postoperatively, T3: before second-stage surgery, and T4: 1 year after loading), the results of bone augmentation using GBR with resorbable membranes and GBR with digital titanium meshes were assessed (Fig. 1). To evaluate both the bone augmentation protocols comprehensively, volumetric stability and complication rates were included as secondary parameters.

## Results

### Demographics

A total of 40 patients (18 men and 22 women) with a mean age of (38.18 ± 13.19) years were enrolled in this clinical study and treated according to the procedure described under ''Materials and methods'' (Fig. 1). Sixty-five sites underwent implant placement and bone augmentation procedures. There were 20 patients and 34 sites in the GBR with the resorbable membrane (RG) group and 20 patients and 31 sites in the GBR with the titanium mesh (Ti-G) group. Sixty implants were placed in the maxilla and five implants in the mandible. Detailed information on implant placement and location is shown in Supplementary Table S1. Among the patients, 7 (17.5%; RG group: 4, Ti-G group: 3) smoked less than 5 cigarettes per day, and 33 (82.5%) were non-smokers. Nine (22.5%; RG group: 5,“ Ti-G group: 4) patients had chronic periodontitis (Patients had undergone periodontal treatment before the implant treatment.), and 31 (77.5%) had no periodontal disease.

### Surgical complications

None of the implants failed during the entire treatment process, and the implant survival rate was 100%. Five patients developed postoperative complications, and the overall complication rate was 12.5% (5/40). In the RG group, one patient developed peri-implant mucositis 8 months after loading, and another patient developed peri-implantitis 13 months after surgery. The incidence of postoperative complications was 10% (2/20), and these two patients did not experience serious bone destruction after timely laser treatment.^25^

In the Ti-G group, titanium mesh exposure occurred at two sites (two patients): one at the distal mid-vertical incision line and the other at the apex of the alveolar ridge incision; both were small-scale late exposure, and the overall titanium mesh exposure rate was 10% (2/20). We removed the exposed parts or the possible sharp edges of the titanium mesh and applied topical minocycline hydrochloride to the incision to promote soft tissue healing. After removal of the titanium mesh in the second stage of surgery, the quality of the underlying new bone remained good. Further, one patient developed peri-implant mucositis at 3 months after loading; however, no significant destruction of the peri-implant bone occurred after timely intervention. Based on the above findings, the incidence of postoperative complications in the Ti-G group was 15% (3/20).

### Bone augmentation outcomes

**Fig. 2:**
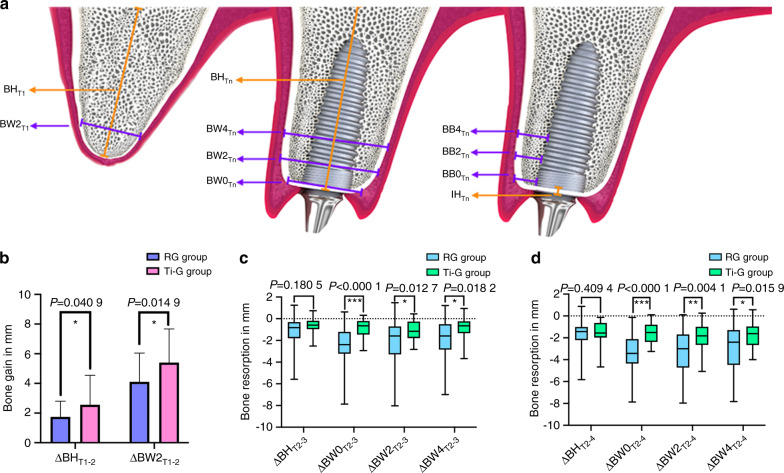
Changes of the alveolar bone. **a** Schematic diagram for the radiographic evaluation (*n* = 1, 2, 3, and 4 represent T1, T2, T3, and T4, respectively). **b** Vertical and horizontal bone augmentation outcomes after surgery. Statistical analysis: *P < 0.05. **c** Vertical and horizontal alveolar bone resorption during the 6-month healing period. Statistical analysis: *P < 0.05, ***P < 0.001. **d** Vertical and horizontal alveolar bone resorption from T2 to T4. Statistical analysis: *P < 0.05, **P < 0.01, and ***P < 0.001

For both the RG group and the Ti-G group, good postoperative bone augmentation was obtained both horizontally and vertically (Supplementary Fig. S1). Based on cone-beam computed tomography (CBCT) measurements (Fig. 2a), we found that the RG group was able to achieve a postoperative augmentation of 1.75 mm ± 1.06 mm in the vertical direction (ΔBH_RG1-2_) and an average increase of 4.10 mm ± 1.95 mm in the horizontal direction (ΔBW2_RG1-2_). The same excellent bone augmentation results could be seen in the Ti-G group: vertically, the alveolar bone increased by a mean of 2.56 mm ± 1.98 mm (ΔBH_Ti1-2_); horizontally, the alveolar bone increased by a mean of 5.41 mm ± 2.26 mm (ΔBW2_Ti1-2_), and all these parameters were statistically different from the RG group (Fig. 2b).

### Changes of the alveolar bone

Resorption of alveolar bone is an important reference index for assessing the stability of bone grafts; therefore, we observed the amount of alveolar bone resorption at the two-dimensional level based on imaging measurements. At T_3_, in the Ti-G group, the values of △BW0_Ti2-3_, △BW2_Ti2-3_, and △BW4_Ti2-3_ were statistically different from the RG group (Fig. 2c). This also indicated that the RG group underwent substantial resorption of bone grafts during the 6-month healing period, while the presence of titanium mesh provided a more stable osteogenic environment for the bone defect area. One year after the Ti-G group was loaded (T_4_), the three parameters (△BW0_Ti2-4_, △BW2_Ti2-4_, and △BW4_Ti2-4_) were still statistically different from the RG group (Fig. 2d). These measurement results also showed that the stable osteogenesis space provided by the digital titanium mesh in the early stage could still have an impact after 1 year of loading.

### The height and thickness of labial bone

**Fig. 3:**
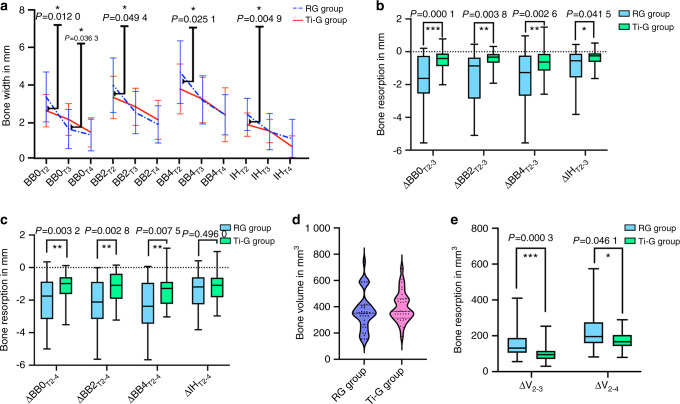
Changes of labial bone and three-dimensional volume at each time point. **a** The height and thickness of the remaining labial bone at each time point. Statistical analysis: *P < 0.05. **b** Labial bone resorption during the 6-month healing period. Statistical analysis: *P < 0.05, **P < 0.01, and ***P < 0.001. **c** Labial bone resorption from T2 to T4. Statistical analysis: **P < 0.01. **d** Bone augmentation volume of each tooth. **e** Bone resorption volume of each tooth. Statistical analysis: *P < 0.05 and ***P < 0.001

Adequate encapsulation of the labial bone determines the long-term stability of the implant; therefore, we also need to evaluate the thickness of the labial bone at different time points. To visualize the changes in the labial bone more clearly, we plotted the thickness of the labial bone as a line graph. Fig. 3a shows that all sites on the labial side of the RG group are thicker than those of the Ti-G group at T2, followed by rapid resorption during the healing period. Finally, the labial bone was thinner in the RG group than in the Ti-G group at the upper part of the implant 1 year after loading. This also explained why the digital titanium mesh avoided a large amount of ineffective bone augmentation. Simultaneously, the stable osteogenic space provided by digital titanium mesh in the early stage also positively influenced the labial bone 1 year after loading. It is worth mentioning that the BB2_Ti4_ was 2.12 mm ± 1.04 mm, which also conforms with our design. Furthermore, we observed that the RG group outperformed the Ti-G group in terms of maintenance of labial bone height above the implant platform.

### Stability of labial bone

In order to assess the stability of the labial bone, we also analyzed its resorption. After a 6-month healing period (T_3_) in the Ti-G group, the labial bone resorption of △BB0_Ti2-3_, △BB2_Ti2-3_, △BB4_Ti2-3_ and △IH_Ti2-3_ were statistically different from the RG group (Fig. 3b). This demonstrated that the digital titanium mesh provided adequate protection for the bone grafts during the healing period, ensuring the stability of the labial bone, and only a small amount of resorption occurred.

Similar results could be observed at T_4_, the Ti-G group had less labial bone resorption than the RG group, △BB0_Ti2-4_, △BB2_Ti2-4_ and △BB4_Ti2-4_ were all statistically different from the RG group (Fig. 3c). This also showed that the stable osteogenesis space and reasonable augmentation range provided by the digital titanium mesh in the early stage had a significant impact on the long-term stability of the labial bone.

### Volumetric stability assessment

**Fig. 4:**
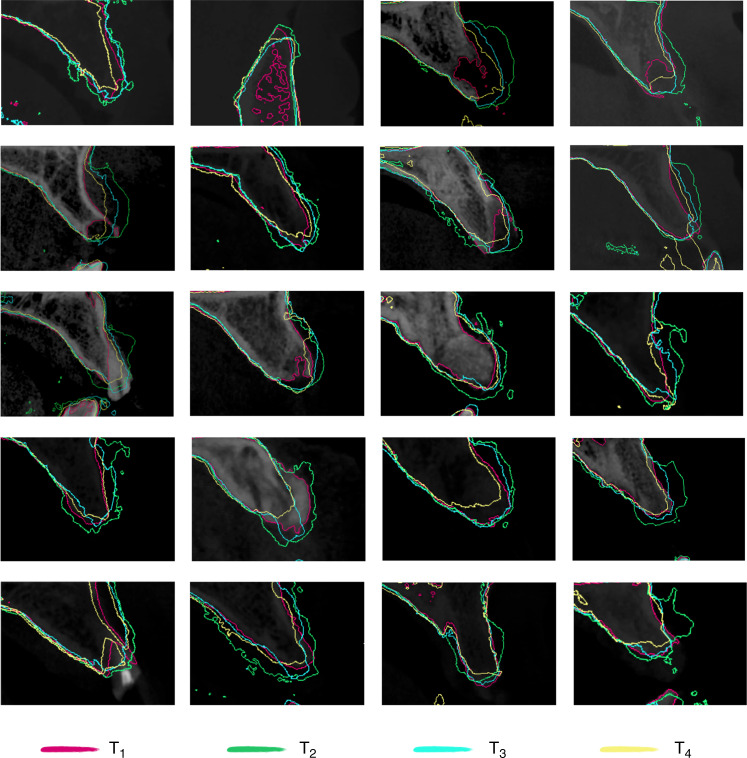
Superimposed radiographic images showing the bone resorption in RG group. Red line, bony profile before surgery; green line, bony profile after surgery; blue line, bony profile before second-stage surgery; and yellow line, bony profile 1 year after loading

**Fig. 5:**
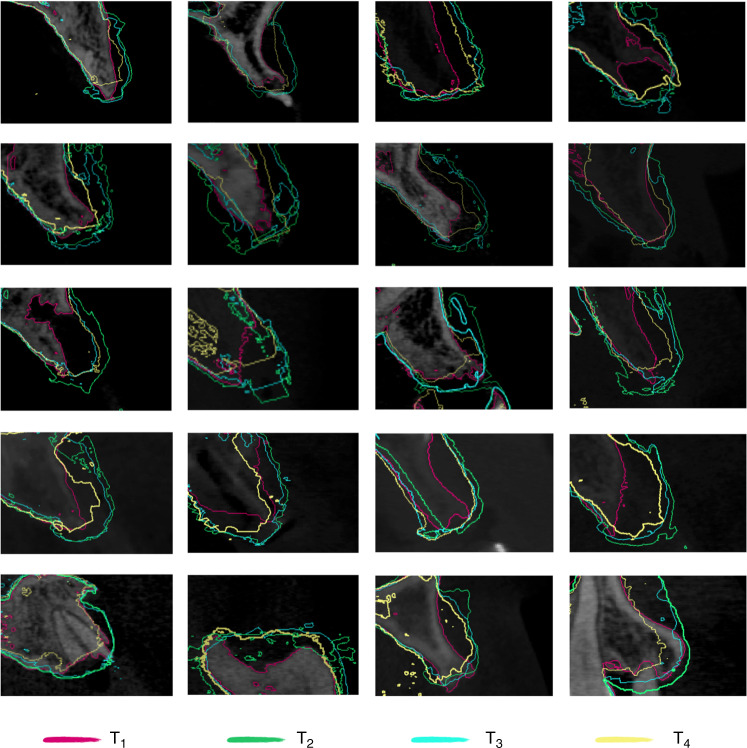
Superimposed radiographic images showing the bone resorption in Ti-G group. Red line, bony profile before surgery; green line, bony profile after surgery; blue line, bony profile before second-stage surgery; yellow line, bony profile 1 year after loading

To provide a more comprehensive assessment of bone volume resorption at the three-dimensional level, Boolean operations was used to calculate the difference in bone volume at different time points. For the RG group, the bone augmentation volume of each tooth (ΔV_RG1-2_) was 351.29 mm^3^ (275.29, 409.74) after the surgery (Fig. 3d); the resorption volume of each tooth was 131.51 mm^3^ (108.48, 185.03) after a 6-month healing period (△V_RG2-3_); after 1 year of loading, the resorption volume of each tooth (△V_RG2-4_) was 195.86 mm^3^ (164.99, 267.12). The Ti-G group obtained (ΔV_Ti1-2_) 365.37 mm^3^ (330.32, 460.00) postoperatively, which was not statistically different from the RG group; the resorption volume of each tooth was 94.93 mm^3^ (71.11, 116.09) after the healing period (ΔV_Ti2-3_), which was statistically different from the RG group; after 1 year of loading, the resorption volume of each tooth (△V_Ti2-4_) was 178.76 mm^3^ (145.19, 220.71), which was statistically different from the RG group (Fig. 3e). The above results and superimposed sagittal radiographic images illustrated that the Ti-G group was superior to the RG group in maintaining bone grafts stability at the three-dimensional level (Figs. 4 and 5).

### Histologic assessment

**Fig. 6:**
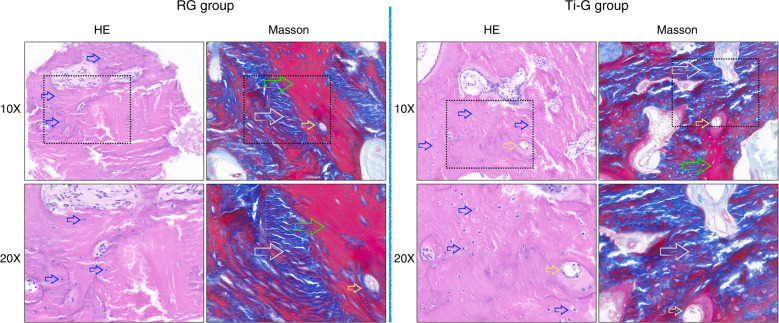
Observation of osteogenesis through histological specimens. Hematoxylin-eosin staining and Masson staining of the alveolar bone at 10× and 20× magnification (blue arrow: bone lacuna, yellow arrow: blood vessel, green arrow: muscle fiber, gray arrow: collagen fiber, and black box: the viewing range of the 20× microscope). In hematoxylin-eosin staining, both RG group and Ti-G groups showed a large amount of new bone formation and a large number of bone lacunas. In Masson staining, more blue areas were seen in the Ti-G group than in the RG group, which confirmed that a large number of collagen fibers were formed in the osteogenic space of the Ti-G group

On Observing by hematoxylin-eosin staining, both RG group and Ti-G group were seen to have a large amount of new bone formation, and bone lacuna existed in it (Fig. 6). In addition, small vascular structures within the new bone tissue could be observed, which indicated that good vascularization results were obtained in the bone augmentation area. In Masson staining, more blue areas showed in the Ti-G group than in the RG group, which confirmed that a large number of collagen fibers were formed in the osteogenic space of the Ti-G group (Fig. 6). The presence of red areas in the RG group demonstrated a certain amount of muscle fiber ingrowth. This might also indicate that the digital titanium mesh was able to provide a purer osteogenic space than the resorbable membrane. Similarly, a good blood supply to the regenerated bone tissue was still observed in the Masson staining.

## Discussion

In this retrospective study, the Ti-G group showed significantly less resorption in the vertical and horizontal directions of the alveolar bone at the two-dimensional level, before the second-stage surgery as well as after 1 year of loading compared with the RG group. This discovery showed that the digital titanium mesh could provide a stable osteogenic space in the early stage, allowing for an undisturbed osteogenic environment, and a large number of bone grafts that could be preserved in the early stage would also have an impact 1 year after loading. To clearly visualize the advantages of the digital titanium mesh in terms of volumetric stability, a statistical analysis of the volume change was performed at each stage. It was found that the amount of resorption in the RG group reached 131.51 mm^3^ (108.48, 185.03) per tooth position before the second-stage surgery, while the postoperative bone gain in the RG group was 351.29 mm^3^ (275.29, 409.74) per tooth position, suggesting that the percentage of resorbable membrane covered GBR resorption reached 37.57% (36.63, 43.19) after 6 months of healing. However, this parameter was only 23.40% (16.37, 31.73) in the Ti-G group. To increase the reliability of the results, volume calculations were performed 1 year after loading; the amount of resorption per tooth position was 195.86 mm^3^ (164.99, 267.12) in the RG group after 1 year of loading, with a higher percentage of resorption (56.26%) compared to that in the Ti-G group (44.76%). In a radiographic study, Lin et al. demonstrated that the bone augmentation beyond the bone arch contour resorb to the bone arch during a 6-month healing period.^26^ This suggests that the evidence-based design of the digital titanium mesh in this study was able to systematically and purposefully restore the peri-implant bone volume and avoid significant bone resorption. Along with excellent spatial maintenance, the digital titanium mesh reduced the resorption rate to a great extent while the volume of new bone formation increased.

The bone resorption that may occur during the healing period should be considered while planning a digital titanium mesh-assisted bone augmentation to achieve a good bone arch contour at the bone defect site even after the healing period and to avoid a large amount of ineffective augmentation. In a long-term follow-up study, radiographic measurements were performed in patients for whom L-type titanium meshes were applied, and they found that the labial bone the implant resorbed by 0–0.4 mm during a maximum follow-up period of 41 months.^17^ In another clinical study, after a 6-month healing period, a 1.1 mm resorption occurred in the vertical direction with titanium mesh-assisted bone augmentation.^24^ Buser et al. suggested that there should be at least 2 mm of the bone plate on the labial side of the implant in the esthetic zone to ensure implant stability.^27^ Accordingly, the implant was placed in a reasonable three-dimensional position, the bone plate thickness was restored to 2 mm on the labial side, and then an additional bone augmentation of 0.5 mm was performed to allow for possible bone resorption during the healing period. Furthermore, an additional bone augmentation of 1 mm in the vertical direction was performed in accordance with the above-mentioned theory.^22,28^ This precise bone augmentation, designed using a digital software, is achieved almost perfectly in the area of the bone defect using a digital titanium mesh as a medium. In our previous study, an accuracy of 95.82% (ranging from 88.53–99.15%) was obtained for the actual augmentation after transferring the virtual information to physical information.^22^ Despite the high accuracy achieved postoperatively, it was important to analyze the remaining alveolar bone after 1 year of loading to ensure that the bone resorption around the implant was still according to our plan after healing and loading. It was found that 1 year after loading, the remaining labial bone plate 2 mm apically from the implant platform was 2.12 mm ± 1.04 mm and the remaining bone height above the implant platform was 0.69 ± 0.58 mm in the Ti-G group, which was also consistent with our prediction during the digital bone augmentation design. Although the RG group had more bone at all postoperative labial sites than did the Ti-G group, after substantial bone resorption, the labial bone was thicker at the upper part of implants in the Ti-G group than in the RG group 1 year after loading. This result confirmed that the digital titanium mesh not only could avoid a large number of invalid bone augmentation, but also could fully protect the labiolingual bone. Therefore, at different time points, there are significant differences in the resorption of alveolar bone and labial bone at two-dimensional and three-dimensional levels. While rejecting the null hypothesis, this result also proved that the digital titanium mesh is superior to the resorbable membrane in maintaining the stability of hard tissues after GBR.

One of the disadvantages of using titanium meshes, which is also a major limitation of their application, is titanium mesh exposure that has an incidence of 20–30%.^29–31^ However, in this study, the Ti-G group exhibited a titanium mesh exposure rate of only 10%. Such a low exposure rate was achieved exclusively because the following three key steps in the treatment process: First, while the thickness of the titanium mesh determines its mechanical strength, a thicker titanium mesh is not easy to perform on the bone model and can lead to overstimulation of soft tissues after bone augmentation.^32^ Therefore, a digital titanium mesh was prepared with a thickness of 0.2 mm, which allowed it to produce less stimulation on soft tissues while taking into account its ability to maintain space. In addition, a digital titanium mesh of this thickness has a certain degree of plasticity, which allows the mesh to be adjusted in a timely manner as required during the operation.^33^ Second, the extent of bone augmentation was precisely delineated during the digital design phase, thus avoiding a large number of ineffective bone grafting outside the contour of the bone arch, which produces greater tension on the soft tissues.^26^ This was the most obvious difference from the traditional titanium mesh. Third, a releasing subperiosteal incision was used in order to achieve initial closure of the tension-free wound, preventing high soft tissue tension on the surface of the digital titanium mesh. Based on these modifications, only two cases of late exposure occurred in the Ti-G group in this study, which is lower than the exposure rates reported by the majority of previous studies (Uehara et al.: 60%; Miyamoto et al.: 36%; and Hartmann et al.: 25%.).^31,34,35^

In summary, the core of the digital titanium mesh used in this study is an evidence-based digital bone augmentation design. Due to this, the application of digital titanium mesh results in less bone resorption, more than 2 mm of the labial bone plate after loading for 1 year, and a low titanium mesh exposure rate. For patients with bone defects that meet the indications, the use of a digital titanium mesh instead of a resorbable membrane not only provides a pure osteogenic space during the bone formation phase, but also allows for good bone encapsulation of the implant after the healing period or after restoration. Moreover, the choice of digital titanium mesh as a treatment option will not increase the trauma and burden of patients. It is important to note that the prerequisite for achieving good clinical results is the placement of the implant in a correct three-dimensional position. Incorrect placement of the implant leads not only to incorrect designing of the digital bone augmentation but also to a significant amount of bone resorption around the implant, ultimately leading to implant failure. Therefore, the digital guides were used to assist in the placement of the implant in all patients. Although the digital titanium meshes demonstrated more stable bone preservation and lower bone resorption at 1 year after loading compared to the resorbable collagen membranes, our group plan to follow the patients further to evaluate the amount of resorption and bone preservation over an extended period of time to verify the reliability of this study.

## Materials and methods

### Study participants

This retrospective clinical study was conducted using the data of patients who sought implant therapy between January 2016 and March 2021 at the Department of Oral Implantology, West China University, Sichuan, China. The participants were divided into two groups: (1) RG group and (2) Ti-G group. A total of 40 patients (65 implant sites) were enrolled. All patients had single or multiple missing teeth and were looking forward to restoring esthetics, articulation, and mastication with implant restorations. Inclusion criteria were as follows: (1) at least 18 years of age, (2) the missing tooth site was a Class 2/4 and 3/4 bone defect (according to Terheyden classification),^22^ (3) reconstruction of the alveolar bone was performed using a resorbable membrane and a digital titanium mesh, (4) healthy adjacent teeth and periodontium, and (5) good general health. The exclusion criteria were as follows: (1) presence of a systemic disease, (2) very poor oral hygiene, (3) extremely severe bone defects, (4) heavy smoking, and (5) pregnant women.

The study was conducted in accordance with the Helsinki Declaration of 1975 as revised in 2000 and the protocol. This retrospective study was approved by the Ethics Committee of Sichuan University (approval number: WCHSIRB-D-2019-101), and all included patients provided written informed consent. All surgical procedures mentioned were performed by an experienced implantologist.

### Digital design protocol

Before the surgery of Ti-G group, the three-dimensional position of the implant was digitally designed to avoid incorrect placement that could affect the treatment results. A digital smile design of the patient’s face and intraoral photographs were collected; a diagnostic wax pattern was created according to the digital smile design, which was then positioned in place of the missing tooth and scanned intraorally (TRIOS 3, 3Shape, Copenhagen, Denmark). The preoperative (T1) DICOM data and intraoral scan data were finally superimposed in 3shape (Copenhagen, Denmark), and the implants were placed in a reasonable three-dimensional position, and then the fully-guided implant plate was printed.

To fabricate a digital titanium mesh, for patients in the Ti-G group, a digital bone augmentation design (Exocad, Darmstadt, Germany) was created after determining the three-dimensional position of the implant. It is well documented that titanium mesh-assisted GBR results in horizontal and vertical resorption of approximately 0.5-and 1- mm, respectively, at approximately 6 months after bone augmentation. Therefore, after designing the implant 3–4 mm below the ideal gingival margin, a 0.5 mm excess bone augmentation was performed on the labial side to ensure a 2.5 mm bone plate thickness, and a 1 mm excess bone augmentation was performed vertically to compensate for possible bone resorption during the postoperative period.^24,36–38^ After the virtually reconstructed bone model was three-dimensionally printed, a titanium mesh (Biomet, Florida, USA) was performed on the model. The final autoclaved titanium mesh was then prepared for intraoperative use (Fig. 1).

### Surgical procedures

On the day of the implant surgery, patients were asked to rinse their mouth with 0.2% chlorhexidine to disinfect; thereafter, local infiltration anesthesia was administered using 4% articaine in the operative area (Supplementary Figs. S2a and S3a). To avoid possible secondary gingival papilla recession after the surgery, a vertical incision was used at the angle of the distal line of the adjacent teeth.^39^ The full-thickness soft tissue flap was turned over to fully expose the bone defect area and to remove any granulation tissue that may be present (Supplementary Figs. S2b and S3b). After the digital implant guide was positioned in the patient’s mouth and the implant socket was prepared according to the guidelines, and the implant placement was completed (placement torque > 20 N-CM) (Supplementary Figs. S2c, S3c, and S3d). This was followed by bone augmentation.

For the RG group, a mixture of deproteinized bovine bone mineral (Bio-Oss, Wolhusen, Switzerland) and autogenous bone and blood was used to cover the bone defect (Supplementary Fig. S2d), a resorbable barrier membrane (Bio-gide, Wolhusen, Switzerland) was used to cover the surface, and the resorbable barrier membrane was stabilized and fixed using resorbable sutures (Supplementary Fig. S2e). For the Ti-G group, a mixture of deproteinized bovine bone mineral, autogenous bone, and blood was filled in the inner side of the digital titanium mesh and in the area of bone defect (Supplementary Figs. S3e and S3f). The preoperatively designed titanium mesh was then held precisely in the area of the bone and fixed with absorbable sutures or titanium screws (Supplementary Figs. S3g and S3h). Finally, tension-free incision closure was completed (Supplementary Figs. S2f and S3i).

CBCT was performed after the operation (T2), and an ice bag was applied locally. To prevent postoperative infection and swelling, patients were prescribed dexamethasone and amoxicillin according to guidelines. The patients were closely followed, and sutures were completely removed 10–14 days after surgery. At 6–8 months after the implant surgery (T3), the patients underwent a second-stage surgery, where the cover screw or titanium mesh was removed through an auxiliary incision at the top of the alveolar ridge.^22^

### Follow-up visits

Patients were assessed at T1, T2, T3, and T4. CBCT was performed at each visit using the same projection parameters and conditions (Fig. 1). Patients were examined for soft tissue swelling, inflammation, or dehiscence to determine the presence of intraoral complications such as infection or titanium mesh exposure and were provided with oral hygiene education and necessary treatment. In this study, the implant survival rate was evaluated according to the criteria for successful implant treatment established by Papaspyridakos.^40^

### Radiographic evaluation

After obtaining CBCT and DICOM data at T1, T2, T3, and T4 (Morita, Kyoto, Japan), we evaluated the alveolar bone and peri-implant bone mass. In order to observe the changes in alveolar bone more meticulously, we superimposed the DICOM data at different time points, and finally measured the changes in the alveolar bone at different levels (Figs. 4 and 5). We defined the following reference lines to achieve optimal measurement repeatability (*n* = 1, 2, 3, and 4 represent T1, T2, T3, and T4, respectively) (Fig. 2a):

BW2_T1_: width of the alveolar bone 2 mm apically from the alveolar crest before surgery.

BH_T1_: height of the alveolar bone as measured on the sagittal plane of the implant axis before surgery.

BB0_Tn_: thickness of labial/buccal plate at the implant platform at Tn.

BB2_Tn_: the thickness of labial/buccal plate 2 mm apical to the implant platform at Tn.

BB4_Tn_: thickness of labial/buccal plate 4 mm apical to the implant platform at Tn.

IH_Tn_: height of labial bone above the implant platform at Tn.

BH_Tn_: height of the alveolar bone was measured on the sagittal plane of the straight line through the implant axis.

BW0_Tn_: width of the residual alveolar bone at the implant platform at Tn.

BW2_Tn_: width of the residual alveolar bone 2 mm apical to the implant platform at Tn.

BW4_Tn_: width of the residual alveolar bone 4 mm apical to the implant platform at Tn.

The above indicators were measured three times by the same researcher, and the average value was obtained. In order to show the effect of bone augmentation, △BW2_T1-n_ was used to represent the horizontal bone augmentation at Tn, and △BH_T1-n_ was used to represent the vertical bone augmentation at Tn; to show bone resorption, the △ symbol was used to indicate the changes in various parameters at different time points (for example, △ BB0_T2-3_ represented the resorption value of the buccal plate at the implant platform from T2 to T3. △ BW4_T2-4_ represented the resorption value of the residual alveolar ridge width 4 mm apically from the implant platform from T2 to T4; Δ BH_T3-4_ represented the change in alveolar height 1 year after loading compared with that of before the second surgery).

### Volumetric stability assessment

To analyze whether the difference in the spatial maintenance ability of these two barrier membranes had an impact on bone augmentation, the changes in bone volume were analyzed. The DICOM data from different time points were imported into a digitizing software (Mimics 21.0, Leuven, Belgium), and the jaws were reconstructed and exported as STL format files. The alveolar bone was superimposed using the anatomical marker points on the bone surface and teeth at different time points (Figs. 4 and 5). The volume change (△V) was obtained at different time periods using Boolean operations (e.g., △V_2-4_ represented the bone resorption volume 1 year after loading compared to the postoperative volume).

### Histologic assessment

In the second-stage surgery, excess bone debris from the patient’s surgical area was collected and hematoxylin-eosin staining and Masson staining were performed on the tissue samples. The bone regeneration results of the RG group and Ti-G group were evaluated by observation of the stained samples.

### Statistical analysis

All data were entered into GraphPad Prism software version 9.0.0 (GraphPad software Inc., San Diego, USA) for statistical analysis. The data distribution was observed by plotting Q-Q plots and analyzing the homogeneity of variance. If the measured data obeyed a normal distribution and the variance is basically the same, the data were expressed using mean ± standard deviation (SD) and the parametric test (Student’s *t*-test) was applied. If the above conditions were not met, the data were expressed using the median (lower quartile, upper quartile) and a nonparametric test (Mann–Whitney *U* test) was applied. *P-*value < 0.05 was considered to be a possible difference.

## Supplementary information

Supporting Information
