# Supplementary material for: Hard tissue stability after guided bone regeneration: a comparison between digital titanium mesh and resorbable membrane
Source: Int J Oral Sci. 2021 Nov 16;13:37. doi: 10.1038/s41368-021-00143-3 (PMC8594427; doi:10.1038/s41368-021-00143-3)
Supplement: Supplementary file 1 — Supporting Information [file 41368_2021_143_MOESM1_ESM.docx]

**Supporting Information:**

**Hard tissue stability after guided bone regeneration: a comparison between digital titanium mesh and resorbable membrane**

**Running Title:** Digital titanium mesh for hard tissue stability

**Authors**

Songhang Li^1^, Junyi Zhao^1^, Yu Xie^1^, Taoran Tian^1^, Tianxu Zhang^1^, Xiaoxiao Cai^1*^.

**Affiliations:**

^1^ State Key Laboratory of Oral Diseases & National Clinical Research Center for Oral Diseases & Department of Implant dentistry, West China Hospital of Stomatology, Sichuan University, Chengdu, China.

**E-mail:**

Songhang Li: [dentistbruce@163.com](mailto:dentistbruce@163.com)

Junyi Zhao: [zhaojunyi98@foxmail.com](mailto:zhaojunyi98@foxmail.com)

Yu Xie: [scuXY1997@163.com](mailto:scuXY1997@163.com)

Taoran Tian: [tiantrcd@163.com](mailto:tiantrcd@163.com)

Tianxu Zhang: [1718913442@qq.com](mailto:1718913442@qq.com)

**Corresponding author:**

^*^Xiaoxiao Cai, State Key Laboratory of Oral Diseases & National Clinical Research Center for Oral Diseases & Department of Implant dentistry, West China Hospital of Stomatology, Sichuan University, Chengdu, China.

Email: [xcai@scu.edu.cn](mailto:xcai@scu.edu.cn)

Table of Contents

Table S1. Detailed information on implant placement and location.

Figure S1. The height and width of the remaining alveolar ridge one year after loading.

Figure S2. Surgical procedures of the RG group.

Figure S3. Surgical procedures of the Ti-G group.

Table S1. Detailed information on implant placement and location.

|  | Upper jaw  (RG group/ Ti-G group) | Lower jaw  (RG group/ Ti-G group) | Total |
| --- | --- | --- | --- |
| Central incisors | 26 (15/11) | 1 (1/0) | 27 |
| Lateral incisors | 22 (15/7) | 1 (1/0) | 23 |
| Canines and premolars | 12 (1/11) | 1 (1/0) | 13 |
| Molars | 0 | 2 (0/2) | 2 |
| Total | 60 | 5 | 65 |


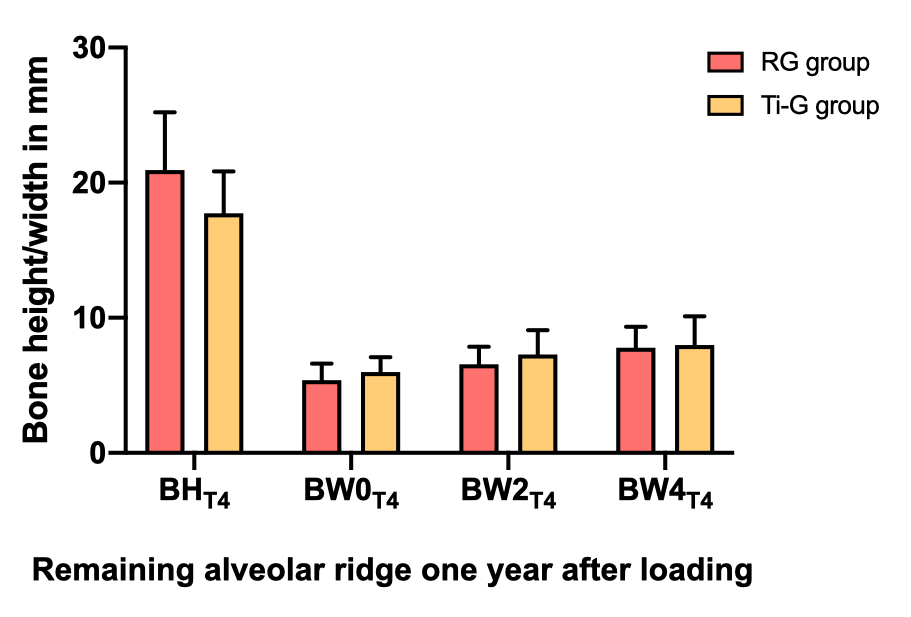


Figure S1. The height and width of the remaining alveolar ridge one year after loading.


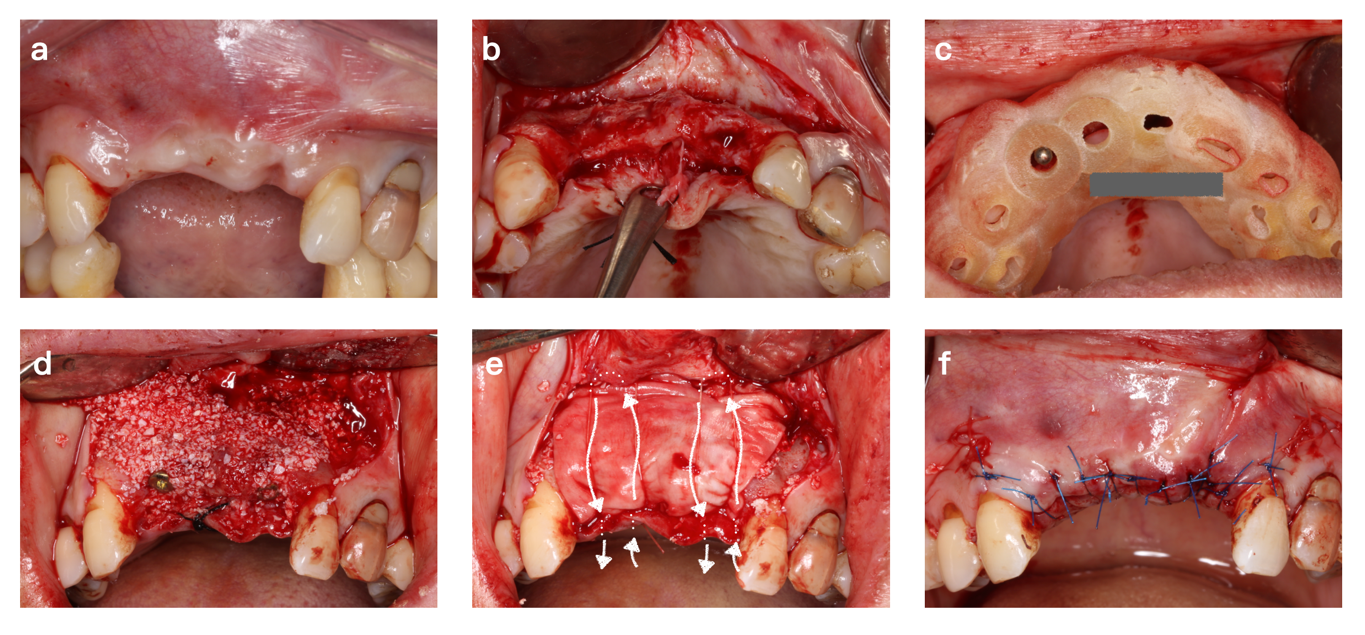


Figure S2. Surgical procedures of the RG group. a. Preoperative intraoral condition. b. Fully expose the bone defect area. c. Implant socket preparations and implant insertion. d. The mixture of bone grafts filled the bone defect area. e. The resorbable barrier membrane was stabilized and fixed with resorbable sutures. f. Tension-free incision closure was completed via single interrupted sutures.


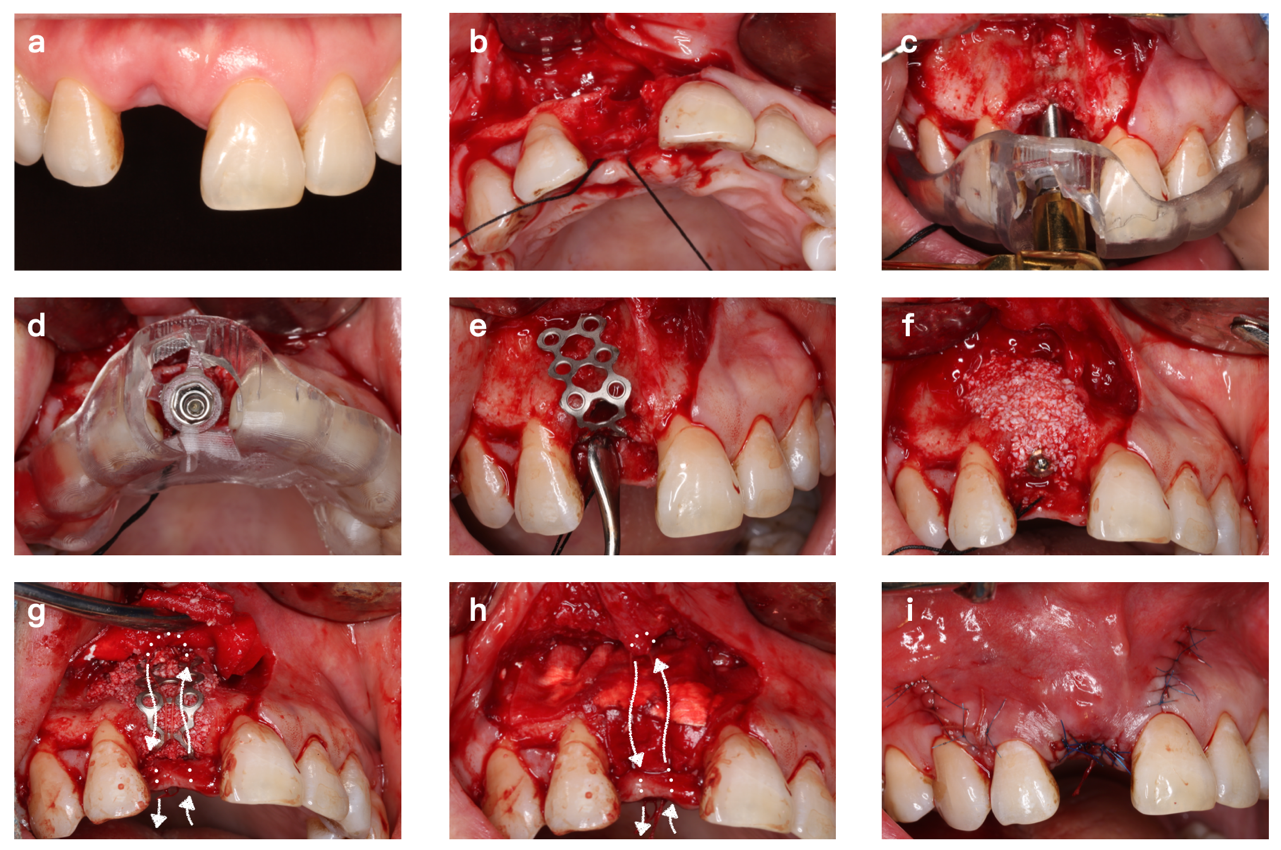


Figure S3. Surgical procedures of the Ti-G group. a. Preoperative intraoral condition. b. Fully expose the bone defect area. c. Implant socket preparations. d. Implant was placed in the ideal three-dimensional position. e. The bone augmentation design was transferring to the digital titanium mesh and application to the bone defect. f. Bone grafts were filled in the inner side of the digital titanium mesh and in the area of bone defect. g. Fixation method when using resorbable sutures to fix digital titanium mesh. h. Surgical sites were covered with a collagen membrane. i. Tension-free incision closure was completed via single interrupted sutures.
